# The Social Determinants of Subjective Cognitive Decline among Adults Age 45 and Over: Implications for Action

**DOI:** 10.1093/geroni/igaf122.2470

**Published:** 2025-12-31

**Authors:** Iftekhar Amin, Syeda Jesmin

**Affiliations:** University of North Texas at Dallas, Dallas, Texas, United States; University of North Texas at Dallas, Dallas, Texas, United States

## Abstract

**Objective:**

Cognitive decline- often the first sign of dementia or Alzheimer’s disease- is the gradual deterioration of one’s ability to remember, learn new things, or make everyday decisions. Among older adults, subjective cognitive decline (SCD) is associated with age-related changes in the brain, injuries, substance use, and mental illnesses. Early detection and diagnosis of SCD have been prioritized in the Healthy People 2030 objectives. There is evidence that socioeconomic status is associated with cognitive decline, however, research is limited on the social determinants of SCD.

**Methods:**

We used data from the 2023 Behavioral Risk Factor Surveillance System (BRFSS). Logistic regression models have been used to examine the association between SCD and a range of social determinants among older adults aged 45 and over.

**Results:**

Several factors from the five domains of social determinants were significant predictors of SCD. Men (OR 2.9, 95% CI = 2.1, 2.6), individuals without a college degree (OR 3.1, 95% CI = 2.2, 3.2), and those with low income (OR 2.7, 95% CI = 1.8, 2.5) were more likely to report SCD. With each additional year of age, the likelihood of SCD increased (OR 1.2, 95% CI = 1.1, 1.6). People reporting isolation (OR 3.6, 95% CI = 2.9, 4.1) and lower levels of social support (OR 4.2, 95% CI = 3.3, 4.9) were more likely to experience SCD.

**Conclusion:**

Findings highlight the importance of increasing SCD interventions for men, individuals with low income and less education, and those who lack social support and experience isolation.

